# Self-esteem among empowered Saudi women

**DOI:** 10.3389/fpsyg.2026.1904899

**Published:** 2026-09-30

**Authors:** Hanaa Faize A. Mubarak, Dahiya Awad Muqbil Al-anazi, Sarah Alhumaidi S. Alanazi, Asyraf Afthanorhan

**Affiliations:** 1 Department of Social Sciences, College of Arts, University of Ha’il, Ha’il, Saudi Arabia; 2 Operation Research & Management Sciences, Faculty of Business and Management, Universiti Sultan Zainal Abidin (UniSZA), Terengganu, Malaysia

**Keywords:** empowerment, Saudi women, self-esteem, women’s empowerment, women’s leadership

## Abstract

**Introduction:**

Self-esteem increases and decreases in response to successes and failures in the domain in which it is invested. Self-esteem has enormous influence on the mental health of women, affecting their adjustment in various spheres of life. A woman’s level of self-esteem is dependent on whether she is empowered or not, especially in societies that are still new to empowering women such as Saudi Arabia.

**Methods:**

This paper aimed to assess levels of self-esteem among empowered Saudi women. The paper belongs to the descriptive studies category, used the social survey method for a purposive sample of (30) women leaders at the University of Ha’il, and two scales of self-esteem; Rosenberg Self-esteem (RSES) and Global Self-esteem (GSE). Using the following statistical analyses: correlation coefficients, reliability, descriptive statistics, and one-way ANOVA.

**Results:**

The results showed that the level of self-esteem among empowered Saudi women is high on both scales. The most prominent manifestations of their self-esteem according to the Rosenberg scale were feeling satisfaction with themselves; feeling they have several good qualities; taking a positive attitude towards themselves; having the ability to do things as well as most other people and feeling that they are people of worth on an equal plane with others. The most prominent manifestations of their self-esteem according to the Global Self-esteem scale were feeling pleased with their achievements; believing in themselves; feeling satisfied with their qualities and being comfortable with who they were. There are no statistically significant differences in the levels of self-esteem of empowered Saudi women according to type of scale, age and leadership period. Whereas there were is statistically significant differences in the levels of self-esteem of empowered Saudi women on the (RSES), while there were on the (GSE).

## Introduction

Women’s empowerment has become a central theme in contemporary development agendas, as institutions around the world increasingly integrate it into their policies and programs. At its core, empowerment refers to the process through which women expand their capabilities, redefine their roles, and gain access to opportunities that were previously restricted. It involves enabling women to participate meaningfully in decision-making structures and to access economic resources that enhance their autonomy. Rather than being an additional component of development efforts, empowerment represents a foundational pillar that supports social and economic progress (Seetharaman, 2022). Its dimensions are multifaceted, encompassing leadership, economic participation, political engagement, education, and health (Sharma and Mehta, 2021).

The persistently low level of women’s empowerment carries profound consequences across multiple dimensions. At the individual level, limited autonomy restricts women’s ability to make informed choices regarding education, health, and career development, often resulting in diminished well-being and reduced self-efficacy (Moid and Dixit, 2020). Within family dynamics, insufficient empowerment undermines women’s participation in household decision-making, which can perpetuate dependency patterns and weaken the stability and resilience of family structures (Akhtar et al., 2025). On a broader societal scale, the underutilization of women’s potential constrains economic growth, hinders social equity, and limits the inclusivity of policy-making processes. Addressing these consequences is therefore essential, as strengthening women’s empowerment not only enhances personal and familial outcomes but also serves as a catalyst for sustainable societal development (Houth et al., 2023). This background underscores the significance and rationale of the present study.

In the Saudi context, women’s empowerment is closely linked to broader social transformation and aligns with national priorities such as the Sustainable Development Goals. Although empowerment is associated with higher social status for all genders, women continue to be underrepresented in senior positions. This has prompted national and international initiatives aimed at improving women’s participation in development and enhancing their well-being. Over recent decades, empowerment has increasingly been viewed as a mechanism for improving women’s quality of life at both personal and professional levels (Singh, 2018).

Leadership development among women is grounded in the idea that sustainable change begins with internal growth and self-reflection. Through continuous practice and constructive feedback, women strengthen their leadership competencies and challenge long-standing biases embedded in organizational and societal structures (Sharma and Mehta, 2021).

In Saudi Arabia, empowering women requires deliberate and sustained efforts, particularly given the cultural norms shaped by long-standing patriarchal traditions. Recent national reforms have significantly expanded women’s roles in public life, especially within the framework of Vision 2030, which prioritizes women’s participation in leadership and decision-making. By 2023, women’s representation in senior and middle leadership roles increased to 2.5% compared to 1.6% in 2017, while the private sector recorded approximately 25% female representation in similar positions.

### Historical overview of women’s empowerment in Saudi Arabia (rewritten)

The trajectory of women’s empowerment in Saudi Arabia has evolved through several stages:

*Early in the twentieth century*, empowerment was largely confined to the family sphere, where women’s status was linked to reproduction and age-related customs. Educational opportunities were limited to kuttabs and a few private schools, and economic participation was restricted to traditional, low-income activities (Al-Ghamdi and Halawi, 1988; Al-Qahtani, 1995; Al-Aidan, 2016; Al-Maizar, 2017).

*Between 1960 and 1980*, a major shift occurred with the expansion of girls’ education, improvements in health services, and the entry of women into the teaching profession (Al-Kurdousi, 1996; Al-Qahtani, 2022).

*From 1980 to 1999*, empowerment progressed unevenly. While educational and health opportunities for women continued to grow, the labor market did not expand at the same pace, and cultural resistance to women’s empowerment intensified (Al-Qahtani, 2022).

*Between 2000 and 2015*, empowerment gained momentum through Saudi Arabia’s commitment to international agreements promoting gender equality. Women received national identity cards, gained equal access to scholarships, and began participating in political bodies such as municipal councils and the Shura Council (Al-Quraini, 2020).

*From 2015 onward*, legal reforms accelerated empowerment by removing restrictions such as the requirement for guardian approval, the driving ban, and travel limitations. New laws were introduced to protect women from violence, and more women were appointed to senior positions. Literacy rates improved dramatically, and educational opportunities became nearly equal for all genders (Saudi Press Agency, 2018; General Authority for Statistics (GASTAT), 2017).

*As women increasingly assume leadership roles across sectors*, an important question emerges: does occupying leadership positions influence their self-esteem? *Therefore, this study aimed to* assess levels of self-esteem among empowered Saudi women. Considering also the absence of any previous study addressing the relationship between empowerment and self-esteem.

Self-esteem represents a central component of personality, influencing how individuals perceive their worth and interact with their environment. It encompasses physical, cognitive, emotional, social, and self-efficacy dimensions (Al-Hajri, 2024). High self-esteem contributes to psychological well-being, social adjustment, and the ability to cope with life’s challenges through constructive problem-solving (Al-Namla, 2017).

### Questions

1. What is the level of self-esteem among empowered Saudi women?
2. What are the features of self-esteem among empowered Saudi women?
3. Are there differences in self-esteem among empowered women according to leadership level?
4. Are there differences in self-esteem among empowered women according to leadership period?

## Literature review

Many researchers have examined the empowerment of Saudi women within leadership contexts, reflecting the growing national interest in enhancing women’s roles across sectors. Adel (2022) explored empowerment practices within the Ministry of Hajj and Umrah, while Makhdoum (2020) investigated how administrative empowerment contributes to the professional development of female academic leaders at Taibah University. Similarly, Attia (2023) identified the institutional requirements necessary to strengthen the empowerment of academic leaders at Umm Al-Qura University.

Other studies have focused on broader structural and organizational dimensions. Al-Khal (2022) analyzed the actual status of women’s empowerment in leadership positions within higher education institutions, highlighting existing challenges and proposing strategies for improvement. Al-Azzam (2021) examined academic leaders’ attitudes toward women’s participation in decision-making in alignment with Vision 2030, while Al-Talhi (2023) explored societal perceptions of women’s leadership in higher education. Saqr (2020) discussed women’s participation in managerial leadership roles amid the social and economic changes accompanying Vision 2030.

Further contributions include the work of Al-Shammasi (2024), who studied psychological empowerment among female leaders in government organizations in Riyadh and its influence on proactive behavior. In the healthcare sector, Jrasat and Faraj Zubaidi (2024) identified cultural, personal, and political barriers that hinder women’s advancement into leadership roles, emphasizing the role of leadership training in enhancing decision-making and expanding opportunities.

Despite the breadth of research on women’s empowerment, there remains a noticeable gap regarding the self-esteem of empowered women, particularly those in leadership positions. Existing studies have examined self-esteem in specific contexts rather than within the broader conceptual framework of empowerment. For example, Karimi et al. (2024) demonstrated that empowerment-based counseling using the GATHER model improved self-esteem and reduced stress among mothers of premature infants. Al-Qahtani et al. (2021) explored self-esteem and self-efficacy among academic and administrative staff in Saudi universities, reporting that participants exhibited moderate to high levels of self-esteem, high self-efficacy, and strong overall empowerment. Research on self-leadership also provides relevant insights; Uzman and Maya (2019) found significant positive associations between self-leadership strategies, self-esteem, and life satisfaction.

## Definitions

*Self-esteem* refers to an individual’s overall positive evaluation of the self (Holloway, 2016). Sedikides and Gregg (2003) stated that self-esteem refers to an individual’s perception or subjective appraisal of one’s own self-worth, one’s feelings of self-respect and self-confidence and the extent to which the individual holds positive or negative views about themself. Self-esteem has two interrelated aspects: sense of personal efficiency and sense of personal worth. On the other hand, self-esteem is defined as the summation of beliefs regarding the individual’s self. These beliefs are obtained through socialization, criticism, the looking-glass self, and self-perception; also defined as perceiving and judging one’s abilities and capabilities to determine one’s self-esteem (Buran and Doğan, 2019).

*Women’s empowerment* is “the processes through which women come to know and correct the gender issues that impede their development” (Longwe, 1998). Women’s empowerment is defined as “the process through which women acquire the ability to make strategic life choices in a context where this ability was previously denied to them”. Huis et al. (2017) have proposed a three-dimensional model of women’s empowerment, which posits that the empowerment of women can take place at three distinct levels, namely, (1) the micro-level or personal dimension manifested in their self-efficacy, (2) the meso-level or relational dimension manifested in the bargaining power that women possess and social capital, and (3) the macro-level or societal dimension manifested in gender disparity in human development.

*Women’s leadership*, Eklund et al. (2017) defined leadership as “the enhancement of behaviors, cognitions and motivations to achieve the goals of a collective group or team where all can benefit”. Callahan and Grunberg (2016) identified a conceptual “FourCe-PITO” framework to capture key elements of today’s description of leadership. The Four C’s (“FourCe”), Character, Competence, Context, and Communication, occur across four different psychological levels -Personal, Interpersonal, Team, and Organizational (PITO). Women bring value to the leadership equation, which sheds light on existing views of how to earn and use power, as well as how to have an enriching professional and personal life. The unique values that women bring to the table are fourfold. First, they are not motivated solely by money and status. Second, they manage their career progress based on achieving their own personal competency; they believe they earn rewards based on their accomplishments. Third, relationships are a key part of their success in leadership. Women prefer to work in a collaborative environment and empower others to achieve financial success. Fourth, they tend to create a balanced harmony between work and family (Koland, 2015).

## Methodology

This paper belonged to descriptive research, with the aim of assessing self-esteem among empowered Saudi women using the social survey method. The study relied on a purposive sample of female leaders at the University of Ha’il (department supervisor - college dean), given the relatively limited size of the original population, which consists of 44 women. The study sample included 30 participants, representing 68.2% of the total population, particularly since these were the individuals who responded to the two scales within the designated data collection period (January and February 2025). Using two scales of self-esteem as follows:

1. Rosenberg Self-Esteem Scale (RSES): The Rosenberg Self-Esteem Scale (RSES), developed by Rosenberg (1965), is a widely utilized instrument designed to assess global self-esteem as a unidimensional construct. The scale comprises 10 items rated on a four-point Likert scale ranging from 1 (strongly disagree) to 4 (strongly agree). Of these, five items are reverse scored to control for response bias. Total scores are calculated by summing all item responses, yielding a range from 10 to 40, with higher scores indicative of greater self-esteem.
2. Global Self-Esteem Scale (GSE): The Global Self-Esteem Scale (GSE), as described by Rajlic et al. (2019), consists of six items intended to measure overall self-esteem. Responses are recorded on a six-point Likert scale ranging from 1 (strongly disagree) to 6 (strongly agree). All items are scored in the same direction, with no reverse scoring applied. The cumulative score ranges from 6 to 36, where higher scores denote elevated levels of self-esteem.

The validity of RSES and GSE were tested through Pearson correlation coefficients (0.796**), (0.864**) respectively. Also, the reliability of RSES and GSE were tested through Cronbach Alpha (0.955), (0.955) respectively, they were statistically significant at the level of (0.01). The use of two self-esteem measures is due to emphasis on the results and the multiplicity of items of self-esteem, because they are uni-dimensional measures. One of the two scales, the RSES, has high psychometric efficiency, many studies have been conducted with it in various countries, and it has been translated into more than 25 languages.

## Results and discussion

### Description of the study sample

The results in (Table 1) showed that 50.0% of the respondents were between 31 and 40 years old, 43.3% were between 41 and 50 years old, while 6.7% were between 51 and 60 years old. As for leadership level, it was found that 60.0% of respondents were department supervisors, 36.7% vice-dean of college/deanship, and 3.3% chancellor. Regarding leadership period, it was found there were 40.0% whose leadership period ranged from 1 to 2 years, 30.0% whose leadership period ranged from 2 to 5 years, 23.3% from 6 to 10 years, and 6.7% whose leadership period exceeded 10 years. Hence the diversity of ages, leadership levels and periods.

**Table 1:** Characteristics of the study sample.

No.	Variables	Responses	*F*	%
1	Age	Under 30 years old	–	–
		31–40 years.	13	43.3%
		41–50 years old.	15	50.0%
		51–60 years.	2	6.7%
		Total	30	100%
2	Leadership level	Supervisor of department	18	60.0%
		Vice-dean of college/ deanship	8	26.7%
		Chancellor	4	13.3%
		Total	30	100%
3	Leadership period	1:2 years	12	40.0%
		2: 5 years	9	30%
		6: 10 years	7	23.3%
		More than 10 years	2	6.7%
		Total	30	100%

### Results about Self-esteem among empowered Saudi women

The results in (Table 2) showed a positive trend in the mean general average of the level of self-esteem among empowered Saudi women according to Rosenberg Scale “RSES”(3.3), as the respondents believe that nine of the 10 manifestations of self-esteem were at a high level, and one was at a moderate level. The manifestations with a high level were: “On the whole, I am satisfied with myself” and “I feel that I have a number of good qualities” with a mean of (3.8);"I take a positive attitude toward myself” with a mean of (3.7); “I am able to do things as well as most other people” and “I feel that I am a person of worth on an equal plane with others” with a mean of (3.5); “I could have more respect for myself” with a mean of (3.4);"I feel I do not have much to be proud of” with a mean of (3.2); “I certainly feel useless at times” with a mean of (3.1), and “At times, I think I am no good at all” with a mean of (2.8). Finally, “All in all, I am inclined to feel that I am a failure” scored a moderate mean of (2.4).

**Table 2:** Self-esteem among empowered Saudi women according to Rosenberg Scale (RSES).

Phrases	Frequency					Descriptive statistics	
	Strongly agree	Agree	Strongly agree	Disagree	Total	*x̄*	Level
All in all, I am inclined to feel that I am a failure.	28	30	12	2	72	2.4	Moderate
I take a positive attitude toward myself.	84	27	–	–	111	3.7	High
I feel I do not have much to be proud of.	44	45	6	1	96	3.2	High
I certainly feel useless at times.	48	33	10	2	93	3.1	High
I am able to do things as well as most other people.	80	18	6	1	105	3.5	High
On the whole, I am satisfied with myself	92	21	–	–	113	3.8	High
I feel that I have a number of good qualities.	100	12	–	1	113	3.8	High
At times, I think I am no good at all.	24	45	14	2	85	2.8	High
I could have more respect for myself.	72	27	2	2	103	3.4	High
I feel that I am a person of worth on an equal plane with others.	68	36	–	1	105	3.5	High
Average mean						3.3	High

(1): 0–1.33 = Low; 1.34–2.66 = Moderate; 2.67–4.0 = High.

(2): The “Frequency” columns represent weighted contributions (n × Likert weight).

The results in (Table 3) showed a high mean general average of the level of self-esteem among empowered Saudi women according to Global Self-esteem “GSE”(5.5), as the respondents agreed that all the six manifestations of self-esteem were at a high level, as the following: “In general, I am pleased with my achievements” and “I believe in myself” with a mean of (5.8); “I am satisfied with the qualities I have” and “I am comfortable with who I am” with a mean of (5.7); “I believe that I make valuable contributions” with a mean of (5.6), and finally “I think of myself in positive terms” scored a mean of (4.3).

**Table 3:** Self-esteem among empowered Saudi women according to Global Self-esteem (GSE).

Phrases	Frequency				Descriptive statistics	
	Strongly agree	Mostly agree	Somewhat agree	Total	*x̄*	Level
I think of myself in positive terms.	120	40	40	130	4.3	High
I believe that I make valuable contributions.	114	45	45	167	5.6	High
I am satisfied with the qualities I have.	132	35	35	171	5.7	High
In general, I am pleased with my achievements.	150	25	25	175	5.8	High
I believe in myself.	144	30	30	174	5.8	High
I am comfortable with who I am.	144	20	20	172	5.7	High
Average Mean					5.5	High

(1): Three responses format: strongly disagree, mostly disagree, and somewhat disagree do not appear.

(2): 0–2.0 = Low; 2.1–4 = Moderate; 4.1–6.0 = High.

(3): The “Frequency” columns represent weighted contributions (n × Likert weight).

It is then evident from the results in (Tables 1, 2) that the level of self-esteem among empowered Saudi women is high, whether on the Rosenberg Scale (RSES) or on the Global Self-esteem (GSE). The most prominent manifestations of their self-esteem according to the Rosenberg scale were satisfaction with themselves; feeling they have several good qualities; taking a positive attitude toward themselves; having ability to do things as well as most other people and feeling that they are persons of worth on an equal plane with others. The most prominent manifestations of their self-esteem according to the Global Self-esteem scale were feeling pleased with their achievements; believing in themselves; feeling satisfied with their qualities and being comfortable with who they were.

The results reveal a noteworthy pattern in which both positive and negative item means on the Rosenberg Self-Esteem Scale are elevated, reflecting a state of self-ambivalence among empowered Saudi women. Participants reported high levels of perceived competence, ability, and overall self-satisfaction, while simultaneously expressing strong self-critical sentiments, such as feeling insufficient pride or experiencing moments of worthlessness. Psychological literature explains this coexistence of positive and negative self-evaluations through the distinction between the two dimensions of self-esteem—self-liking and self-competence—as articulated by Tafarodi and Swann (1995). Within the Saudi context, such ambivalence may reflect a transitional phase in which women navigate between traditional social roles that emphasize modesty and self-critique, and emerging leadership roles that foster a sense of capability and achievement. Thus, this pattern should not be viewed as contradictory; rather, it represents an important psychological and cultural characteristic that must be considered when interpreting self-esteem levels among empowered women.

### Results about significance differences between study variables

To address the different measurement scales of the Rosenberg Self-Esteem Scale (RSES; range 10–40) and the General Self-Efficacy Scale (GSE; range 6–36), raw scores were standardized into z -scores prior to analysis. This process centered both scales around a mean of 0 with a standard deviation of 1, allowing for direct comparison. Cross-instrument consistency was then assessed using Pearson correlation analysis alongside a paired-samples t -test on the standardized values as depicted in the following Table.

**Table:** 

Correlations			
		RSES	GSE
RSES	Pearson Correlation	1	0.573^**^
	Sig. (2-tailed)		0.001
	N	30	30
GSE	Pearson Correlation	0.573^**^	1
	Sig. (2-tailed)	0.001	
	*N*	30	30

**Correlation is significant at the 0.01 level (2-tailed).

A Pearson correlation analysis was conducted to evaluate the relationship between self-esteem (RSES) and general self-efficacy (GSE). The results showed a statistically significant, moderate-to-strong positive correlation between the two instruments, r(28)=0.573,p=0.001,N=30 . This indicates that participants with higher self-esteem scores consistently demonstrated higher general self-efficacy, confirming solid alignment across the standardized measures.

Table 4 showed there is significant difference between RSES and GSE as the probability value is 0.017, less than the threshold value of 0.05.

**Table 4:** Significant differences in self-esteem according to scale (RSES) and (GSE).

One Way Anova			Sum of squares	df	Mean square	*F*	Sig.
RSES * GSE	Between Groups	(Combined)	1.939	5	0.388	3.479	0.017
	Within Groups		2.675	24	0.111		
	Total		4.615	29			

Given the ANOVA output (Table 5), it seems that there is no significant difference on either RSES or GSE according to age, as the probability from both outputs are greater than 0.05 (*p*-value = 0.400 and *p*-value = 0.443).

**Table 5:** Significant differences in self-esteem according to age.

One Way Anova		Sum of squares	df	Mean Square	*F*	Sig.
RSES	Between groups	0.303	2	0.152	0.949	0.400
	Within groups	4.312	27	0.160		
	Total	4.615	29			
GSE	Between groups	0.216	2	0.108	0.839	0.443
	Within groups	3.473	27	0.129		
	Total	3.689	29			

Given ANOVA output (Table 6), it seems that there is a significant difference on GSE instruments according to the Leadership level (*p* value = 0.005 < 0.05), whereas there is no significant difference on RSES.

**Table:** 

Multiple comparisons							
LSD							
Dependent variable	(I) Leader level	(J) Leader level	Mean difference (I-J)	Std. error	Sig.	95% Confidence interval	
						Lower bound	Upper bound
RSES	Supervisor	Vice Dean/Dean	0.21667	0.16543	0.201	−0.1228	0.5561
		Chancellor	−0.20833	0.21521	0.342	−0.6499	0.2332
	Vice Dean/Dean	Supervisor	−0.21667	0.16543	0.201	−0.5561	0.1228
		Chancellor	−0.42500	0.23841	0.086	−0.9142	0.0642
	Chancellor	Supervisor	0.20833	0.21521	0.342	−0.2332	0.6499
		Vice Dean/Dean	0.42500	0.23841	0.086	−0.0642	0.9142
GSE	Supervisor	Vice Dean/Dean	0.37269*	0.12941	0.008	0.1072	0.6382
		Chancellor	−0.23148	0.16835	0.180	−0.5769	0.1139
	Vice Dean/Dean	Supervisor	−0.37269*	0.12941	0.008	−0.6382	−0.1072
		Chancellor	−0.60417*	0.18650	0.003	−0.9868	−0.2215
	Chancellor	Supervisor	0.23148	0.16835	0.180	−0.1139	0.5769
		Vice Dean/Dean	0.60417*	0.18650	0.003	0.2215	0.9868

*The mean difference is significant at the 0.05 level.

**Table 6:** Significant differences in Self-esteem according to Leadership level.

One Way Anova		Sum of squares	df	Mean Square	*F*	Sig.
RSES	Between groups	0.522	2	0.261	1.722	0.198
	Within groups	4.093	27	0.152		
	Total	4.615	29			
GSE	Between groups	1.185	2	0.592	6.386	0.005
	Within groups	2.504	27	0.093		
	Total	3.689	29			

Fisher’s Least Significant Difference (LSD) post-hoc comparisons were conducted to evaluate pairwise differences across leadership levels for both self-esteem (RSES) and general self-efficacy (GSE). For RSES, no statistically significant pairwise differences were found among Supervisors, Vice Deans/Deans, and Chancellors ( p>0.05 ).

In contrast, the post-hoc analysis for GSE revealed significant variation across leadership tiers. Supervisors demonstrated significantly higher general self-efficacy compared to Vice Deans/Deans ( MD=0.373,SE=0.129,p=0.008,95%CI[0.107,0.638] ). Similarly, Chancellors scored significantly higher on GSE than Vice Deans/Deans ( MD=0.604,SE=0.187,p=0.003,95%CI[0.222,0.987] ). No significant difference in GSE was detected between Supervisors and Chancellors ( MD=−0.231,p=0.180 ). These findings indicate that Vice Deans/Deans reported lower self-efficacy relative to both operational supervisors and executive leadership.

Given the ANOVA output (Table 7), it seems that there are no significant differences between Leadership Period on RSES or GSE as the probability from both outputs are greater than 0.05 (*p*-value = 0.423 and *p*-value = 0.653).

**Table 7:** Significant differences in self-esteem according to leadership period.

One way Anova		Sum of squares	df	Mean Square	*F*	Sig.
RSES	Between groups	0.463	3	0.154	0.967	0.423
	Within groups	4.152	26	0.160		
	Total	4.615	29			
GSE	Between Groups	0.220	3	0.073	0.549	0.653
	Within groups	3.469	26	0.133		
	Total	3.689	29			

The above showed that there are no statistically significant differences in the self-esteem of empowered Saudi women according to the variables of type of scale, age and leadership period. As for the level of leadership, it was found that there were no statistically significant differences in the self-esteem of empowered Saudi women on the (RSES) scale, while there were statistically significant differences on the (GSE) scale.

The above results are consistent with consideration that self-esteem and self-confidence reflect an individual’s emotional evaluation of their own worth. It helps the evaluation of individuals by their own potential and helps to explore themselves (Poongavanam, 2023). Self-esteem is one of the most important elements that affect overall human behaviors and relationships. It affects a person’s ability to accept responsibilities, to cooperate with others, communicate effectively, to deal with positive and negative emotions, accept challenges, and tolerate stressful life events. The most common features of high high levels of self-esteem in individuals include high personal empowerment, reflected in their self-efficacy. Self-esteem influences the perception of work conditions, decision-making in daily life, personal growth, and coping strategies that a person can choose in different situations. Persons with high self-esteem and self-worth are more likely to recognize stress as an opportunity for personal growth (Al-Qahtani et al., 2021).

Gender, as one of the central attributes of identity, and gender-related traits are significantly linked with self-esteem. The centrality of contingencies of self-worth is also gender-related, showing the stronger dependence of women’s self-esteem on external sources of self-esteem (e.g., family support or others’ approval) compared to the stronger independence of man’s self-esteem. Self-esteem increases and decreases in response to successes and failures in the domain in which their self-esteem is invested. The description of these domains was made within the framework of contingencies of self-worth. The contingency of self-worth is defined as “a domain or cate-gory of outcomes on which a person has staked his or her self-esteem, so that a person’s view of his or her value or worth depends on perceived successes or failures or adherence to self-standards in that domain” (Mandal and Moroń, 2019). Self-esteem is a powerful force within each one of us. It encompasses much more than that innate sense of self-worth which presumably is our human birthright. Self-esteem is defined as: Appreciating my own worth and importance and having the character to be accountable for myself and to act responsibly toward others (Branden, 1992). Self-esteem has enormous influence on the mental health of women. Self-esteem comprises self-worth and self-image, which affects women’s adjustment in various spheres of life (Jan and Ashraf, 2008).

## Conclusion

Self-esteem is considered one of the central constructs in social psychology, and Tafarodi and Swann (1995) provided a well-established theoretical framework for understanding it by distinguishing between two fundamental dimensions that constitute global self-esteem: self-liking and self-competence. The first dimension refers to an individual’s feelings toward the self in terms of worth, acceptance, and satisfaction, whereas the second reflects the individual’s perception of their abilities, competence, and effectiveness in achieving goals. The two scholars demonstrated that, although these dimensions are interrelated, they maintain theoretical independence, and overlooking either one results in an incomplete understanding of the nature of self-esteem. This model is among the most influential in explaining individual differences in confidence and self-satisfaction, and it offers a solid theoretical foundation for analyzing the findings of studies that examine self-esteem across diverse social and cultural contexts.

This dual model aligns closely with the present study, which sought to assess self-esteem levels among empowered Saudi women, particularly in light of the recent social transformations occurring in the Kingdom of Saudi Arabia in the realm of women’s empowerment. Empowerment not only enhances women’s sense of competence and ability to achieve but also strengthens their sense of worth and self-acceptance. Accordingly, the use of the Rosenberg Self-Esteem Scale (RSES) and the Global Self-Esteem Scale (GSE) in this study provided a more comprehensive understanding of the two dimensions identified by Tafarodi and Swann and contributed to explaining how empowerment manifests in a balanced elevation of both self-liking and self-competence among women in leadership positions.

The study’s findings revealed high levels of self-esteem among empowered Saudi women on both scales, consistent with the model proposed by Tafarodi and Swann (1995). The Rosenberg Scale indicated that participants felt satisfied with themselves, perceived themselves as possessing positive qualities, adopted a positive attitude toward their self-concept, and felt competent relative to others—indicators that collectively reflect both self-liking and self-competence. Similarly, the Global Self-Esteem Scale showed that the women felt pleased with their achievements, believed in their abilities, and were comfortable with who they were, clearly reflecting the same two dimensions. These results suggest that empowerment—through opportunities for leadership, decision-making, and achievement—enhances self-competence, while social recognition and leadership status reinforce self-liking, ultimately contributing to an overall elevation in global self-esteem.

The results also indicated no statistically significant differences in self-esteem levels based on age or length of leadership experience, suggesting that empowerment itself—rather than age or tenure—is the most influential factor in strengthening self-esteem among Saudi women. This finding reflects the broader social and cultural shifts underway in the Kingdom, where expanding opportunities for women’s empowerment contribute to greater confidence and satisfaction with their abilities and accomplishments. Although the history of women’s empowerment in Saudi Arabia is relatively recent, this study aimed to assess the self-esteem of empowered female university leaders, and the findings demonstrated notably high levels of self-esteem on both scales. Nevertheless, the researcher believes that further studies with larger samples of empowered women across various fields, as well as unempowered women, are needed to better understand the nuanced differences in self-esteem between these groups.

It is important to note that Saudi women’s self-esteem is influenced by a wide range of social, cultural, and psychological variables, with empowerment representing only one of these factors. Despite the many recent indicators that the Kingdom is moving toward greater empowerment of women, examining the impact of this empowerment on different aspects of Saudi women’s lives—beginning with self-esteem, as in the present study, and extending to social relationships, skills, choices, and resource management—is essential for understanding the profound transformations they are experiencing. Moreover, Arab Muslim women possess a distinct psychological and cultural profile characterized by unique features that warrant in-depth study to pave the way for interpreting the many issues they encounter within rapidly changing social contexts.

Finally, the study conclusion should acknowledge an important limitation that was mentioned in the methodology and merits reiteration here: the relatively small sample size of the current study. The sample consisted of 30 participants, representing 68.2% of the target population. Although this is not a small proportion relative to the original population size, it remains a limited sample in absolute terms. Consequently, caution should be exercised when generalizing the findings, as they may not fully reflect the actual diversity of Saudi society. Female leaders are represented across various professional fields, administrative levels, and degrees of empowerment, each of which may influence self-esteem in different ways. Therefore, further research involving larger and more diverse samples is needed to provide a more comprehensive understanding of self-esteem among empowered Saudi women and to enhance the generalizability of the findings.

## Data Availability

The datasets presented in this study can be found in online repositories. The names of the repository/repositories and accession number(s) can be found at: https://doi.org/10.5281/zenodo.18332683.
